# Changes and Correlations of the Intestinal Flora and Liver Metabolite Profiles in Mice With Gallstones

**DOI:** 10.3389/fphys.2021.716654

**Published:** 2021-08-19

**Authors:** Yang Chen, Qiang Wang, Wenqi Gao, Biao Ma, Dongbo Xue, Chenjun Hao

**Affiliations:** ^1^Department of General Surgery, The First Affiliated Hospital of Harbin Medical University, Harbin, China; ^2^Department of General Surgery, Heilongjiang Provincial Hospital, Harbin, China

**Keywords:** gallbladder stones, intestinal flora, liver metabolic disorders, intestinal liver axis, dysbacteriosis

## Abstract

There is increasing appreciation for the roles of the gut-liver axis in liver and gall diseases. Specific gut microbes are associated with susceptibility to gallstone diseases, while the relationship between intestinal flora and liver metabolism in the formation of gallstones remains unclear. In this study, an experimental group of model mice was given a lithogenic diet, and a control group was given a normal diet. Both groups were fed for 8 weeks. Integrating 16S rRNA gene sequencing and non-targeted metabolomics to explore the impact of the lithogenic diet on intestinal flora and liver metabolism, Spearman correlation analysis reveals the network of relationships between the intestine and liver. Our findings showed that the gut microbiome and liver metabolome compositions of the test group were significantly changed compared with those of the normal group. Through our research, biomarkers of gallstones were identified at the phylum (5), class (5), order (5), family (7), and genus levels. We predicted the function of the differential flora. We analyzed the liver metabolism of mice with gallstones paired with their flora, and the results showed that there were 138 different metabolites between the two groups. The metabolic pathways enriched by these differential metabolites are highly consistent with the functions of the disordered flora. We focused on an analysis of the relationship between deoxycholic acid, asymmetric dimethylarginine, glucosamine, tauroursodeoxycholic acid, and the disordered flora. This provides a basis for the establishment of the intestine-liver axis in gallstone disease. This research provides a theoretical basis for the research and development of probiotics and prebiotics.

## Introduction

Gallstones are very common worldwide and affect 10–20% of the global adult population (Lammert et al., 2016). Recently, due to improved lifestyles and increased consumption of fat- or cholesterol-rich diets, the prevalence of gallstone disease has increased rapidly. Currently, the treatment for gallstone disease remains predominantly invasive; however, cholecystectomy potentially generates health problems, including intestinal dysfunction (Di Ciaula et al., 2019) and even increased colon cancer risk (Chen et al., 2014, 2020; Shabanzadeh et al., 2017). Therefore, future efforts should focus on preventive strategies to prevent the formation of gallstones. Hepatic hypersecretion of cholesterol caused by genetic and dietary factors is the main reason of the formation of cholesterol gallstones. Intestinal factors leading to the formation of cholesterol gallstones include reduced absorption of bile salt and increased absorption of cholesterol. The intestinal flora, as an intestinal factor, plays an important role in the formation of gallstones.

Accumulating evidence suggests that the intestinal flora affects liver and gall diseases by regulating the gut-liver axis (Jia et al., 2019; Lang et al., 2020). Compositional changes in the fecal bacterial microbiome of patients with GSD have been reported in several cross-sectional studies (Wu et al., 2013; Wang et al., 2020). Ruminococcus gnavus is a marker for distinguishing gallstone patients from healthy controls (Wang et al., 2020). Gallstone patients had higher levels of 7alpha-dehydroxylating bacteria than patients without gallstones (Wells et al., 2000). All strains of 7alpha-dehydroxylating bacteria appear to belong to the genus Clostridium (Wells et al., 2000; Begley et al., 2005), and the abundance of Clostridium also increases in mice with gallstones (Wang et al., 2017, 2020). The intestinal flora cooperates with the liver to regulate bile acid and fat metabolism (Sayin et al., 2013), which can in turn contribute to the development of gallstones.

Researchers have collected bile and serum samples from patients with gallstones (Sharma et al., 2017; Molinero et al., 2019; Petrov et al., 2020) to explore the formation of gallstones from the perspective of metabolomics. Patients with gallstones had different bile metabolic profiles compared with individuals without hepatobiliary disease (Molinero et al., 2019). Deoxycholic acid is a metabolite of the genus Clostridium, and high levels of deoxycholic acid in bile feces are associated with an increased risk of cholesterol gallstone disease (Molinero et al., 2019; Wang et al., 2020). Trimethylamine-N-oxide (TMAO) is a microbial-dependent metabolite that increases the expression of abcg5/8 in the liver to increase the secretion of cholesterol in hepatocytes and then promote the formation of gallstones (Chen et al., 2019). The liver is an important organ for bile acid production and metabolism of cholesterol and lipids, which are all related to the formation of gallstones. Therefore, liver metabolomics is an important aspect of our exploration of gallstone formation.

To the best of our knowledge, few studies have examined liver metabolomics in gallstone patients or mice, while no study has examined the association between the intestinal flora, liver metabolomics and gallstones. Therefore, in our study, gallstones in model mice were induced by lithogenic diet (LD), and 16S rRNA gene sequencing and LC/MS-based metabolomics were applied to provide more information on the interplay between the intestinal flora and liver, with the purpose of identifying the possible mechanism of gallstone formation in the flora-gut-liver axis.

## Materials and Methods

### Animal Experiments and Sample Collection

All experimental protocols were approved by the Ethics Committee of Harbin Medical University. Adult male C57BL/6J mice (3 weeks old, Liaoning Changsheng Biotechnology Co., Ltd, Liaoning, China) were housed in a controlled environment (12-h light-dark cycle) in terms of temperature (18–24°C) and humidity (50–60%). Each group was fed the indicated diet and water ad libitum. The mice were given 1 week to adapt to the new environment, followed by 8 weeks of dietary intervention. Twenty mice were randomly divided into the control group (group C) fed a normal diet and the test group (group T) fed a lithogenic diet (LD, containing 1.25% cholesterol and 0.5% cholic acid). After the 8 weeks of dietary intervention, we collected feces at 7 o’clock every morning. We used cotton swabs to stimulate the end of the rectum in mice to promote defecation. We collected mouse feces in sterile tubes and immediately froze them in a −80°C refrigerator. Mice were euthanized after an 8-h fast. To reduce the effects of postmortem delay on liver metabolites, all samples were collected and frozen at −80°C within 10 min after death.

### Gallstone Examination and Liver Histology

To observe the effects of lithogenic diet-induced gallstone phenotypes, gallstone formation was evaluated macroscopically, and gallbladder bile was examined by polarizing light microscopy without a cover slip. The liver was removed and fixed in 10% formalin and then processed and embedded into wax blocks. After sectioning, the liver sections were stained with hematoxylin and eosin (HE) to examine hepatic steatosis.

### DNA Extraction and Intestinal Flora 16S rRNA Sequencing

Total genomic DNA of samples was extracted using the MagPure Soil DNA LQ Kit (Magen, Guangzhou, China). The concentration of DNA was verified with a NanoDrop (Thermo Fisher, United States) and agarose gel electrophoresis. Bacterial DNA was amplified with primers targeting the V3–V4 regions (5′-TACGGRAGGCAGCAG-3′, 5′-AGGGTATCTAATCCT-3′). Amplicon quality was visualized using gel electrophoresis, purified with AMPure XP beads (Agencourt), and amplified for another round of PCR (Bio-Rad, United States). After purification with AMPure XP beads again, the final amplicon was quantified using a Qubit dsDNA assay kit (Life Technologies, California, United States). Equal amounts of purified amplicons were pooled for subsequent sequencing by the Illumina MiSeq platform (Illumina, California, United States) by OE Biotech (Shanghai, China).

Raw sequencing data were in FASTQ format. The raw data were treated and processed using the QIIME software package (version 1.8.0). Clean reads were subjected to primer sequence removal and clustering to generate operational taxonomic units (OTUs) using Vsearch software with a 97% similarity cutoff. The representative read of each OTU was selected using the QIIME package. All representative reads were annotated and BLASTed against the Silva database Version 123 using the RDP classifier (confidence threshold was 70%).

### Liver Metabolomic Analyses

Extraction and sample preparation were performed according to a protocol described previously (Sun et al., 2021). Non-targeted metabolite analysis was performed by OE Biotech (Shanghai, China). A Dionex Ultimate 3,000 RS UHPLC system fitted with a Q Exactive quadrupole Orbitrap mass spectrometer equipped with a heated electrospray ionization (ESI) source (Thermo Fisher Scientific, Waltham, MA, United States) was used to analyze the metabolic profiling in both ESI-positive and ESI-negative ion modes. An ACQUITY UPLC HSS T3 (1.8 μm, 2.1 mm × 100 mm, Waters, United Kingdom) was employed in both positive and negative modes. The binary gradient elution system consisted of (A) water (containing 0.1% formic acid, v/v) and (B) acetonitrile (containing 0.1% formic acid, vol/vol). The initial composition was 95% A and 5% B. Separation was achieved using the following gradient: 5% B, 0.01–2 min; 5–30% B, 2–4 min; 30–50% B, 4–8 min; 50–80% B, 8–10 min; 80–100% B, 10–14 min. The composition was held at 100% B for 2 min; 100–5% B, 15–15.1 min; 5% B, 15.1–16 min. The QC samples were injected at regular intervals (every 10 samples) throughout the analytical run to provide a set of data from which repeatability could be assessed. The acquired LC-MS raw data were analyzed by progenesis QI software (Non-linear Dynamics, Newcastle, United Kingdom). Metabolites were identified by progenesis QI (Waters Corporation, Milford, United States) Data Processing Software.

### Methods of Statistical Analysis

The Wilcoxon rank sum test was applied to assess the significant differences in α diversity between two compared groups, and β diversity was visualized by principal coordinates analysis (PCOA) using weighted Unirac distance matrix data. LEfSe differences between two groups were tested for significance using the Kruskal-Wallis sum-rank test, and biological significance was subsequently investigated using a set of pairwise tests among groups with the Wilcoxon rank-sum test. Finally, linear discriminant analysis (LDA) was used to obtain the final differential species, followed by the Wilcoxon rank-sum test. The metagenomes of the intestinal flora were imputed from 16S rRNA sequences using PICRUSt. The predicted KEGG database^1^ results were counted at three levels according to the Wilcoxon algorithm. Multivariate statistical analysis first uses unsupervised principal component analysis (PCA) to observe the population distribution among samples and the stability of the whole analysis process and then uses orthogonal partial least squares analysis (OPLS-DA) to distinguish the overall differences in metabolic profiles among groups and find the metabolites that are different between groups. In this study, the default 7-round cross validation was applied, with one seventh of the samples excluded from the mathematical model in each round to guard against overfitting. The screening standard for differential metabolites was that the first principal component in OPLS-DA model was VIP>1, *T*-test *p* < 0.05. Pathway enrichment analysis of differential metabolites was performed by using the KEGG database. In addition, hypergeometric tests were used to identify pathway entries that were significantly enriched in significantly differentially expressed metabolites compared with the overall background, and pathways with *p* < 0.05 were considered significantly enriched. The correlation between the gut microbiome and liver metabolome was analyzed using Spearman correlation tests (Student’s *t*-test, *p* < 0.05, | correlation coefficient| > 0.3). All analyses and graphics were performed using QIIME (version 1.8.0), R software (version 3.6.2), and R software (version 3.5.1). All values are presented as the means ± SEM. The results with *p* < 0.05 were considered statistically significant.

## Results

### Gallbladder Lithogenesis Rate and Liver Pathological Changes

**FIGURE 1:**
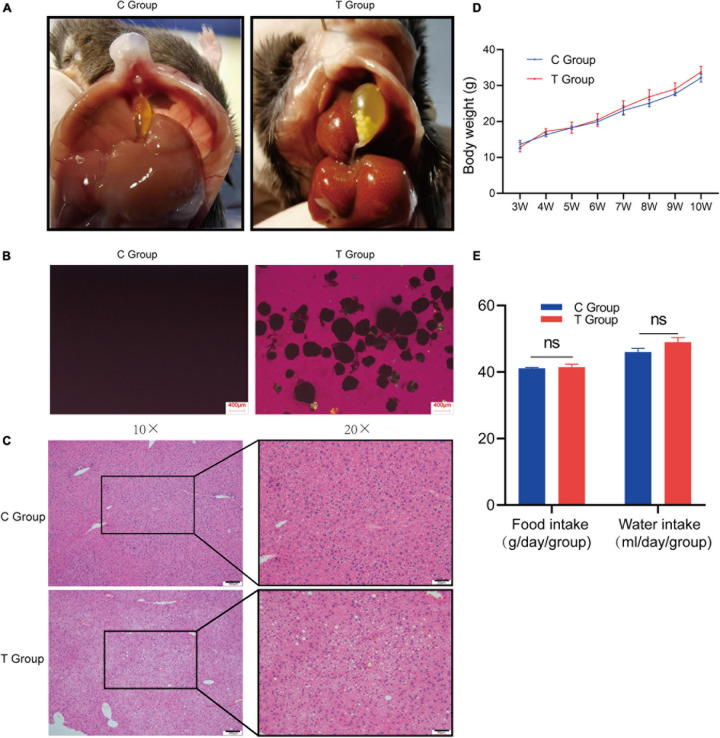
Gallstone formation and liver pathology of C57BL/6J mice after 8 weeks of dietary intervention. **(A)** Macroscopic appearances of the gallbladders of T group and C group mice. **(B)** Polarized light microscopy of gallbladder bile of T group and C group mice. **(C)** Severe steatosis occurred, along with inflammatory cell infiltration and hepatocyte necrosis in the liver cells of the T group, and liver cells were well formed in the C group. **(D)** Changes in body weight of mice from 3 weeks of age to 10 weeks of age. The figure shows that the body weight of the two groups of mice is not statistically significant. **(E)** Calculate the daily food intake and water intake of the two groups of mice. The results showed that there was no statistical difference in the food intake and water intake of the two groups of mice.

After 8 weeks of dietary intervention, as expected, cholesterol gallstones were observed in all mouse gallbladders of the T group, and cholesterol crystals were also confirmed by polarized-light microscopy (lithogenesis rate 100%; Figure 1A), while no cholesterol gallstones or crystals were found in the C group either by gross observation or polarized-light microscopy (lithogenesis rate 0%; Figure 1B). Pathological sections stained with H&E showed that there were larger lipid droplets, lamellar necrosis and inflammatory cell infiltration in the liver cells of the T group mice, but they did not exist in the C group mice (Figure 1C). The results show that mice with gallstones have fatty liver. The two groups of mice did not exhibit any changes in body mass from 3 to 10 weeks (Figure 1D), and the food intake and water intake of the two groups of mice did not change significantly (Figure 1E). This shows that the lithogenic diet does not cause differences in body weight, food and water intake.

### Effects of LD Interventions on Intestinal Flora

The alpha diversity of the intestinal flora in gallstone model mice was significantly reduced. The significant decrease in the Chao1 index of the gallstone group indicates that the gut microbial abundance of gallstone mice was significantly reduced (Supplementary Figure 1A), but the significant increase in the goods coverage index indicates that the biodiversity was reduced (Supplementary Figure 1B). The results of the animal model are consistent with the changes in the intestinal flora of patients with gallstones. The species accumulation curve tends to flatten gradually, indicating that the sampling is sufficient, and the samples can reflect the richness of species (Supplementary Figure 1C). The rank abundance curve reflects the abundance and uniformity of species in the sample (Supplementary Figure 1D). Compared with the control group, the beta diversity of the intestinal flora of gallstone model mice was significantly different.

Beta diversity is the degree of diversity between biological environments, that is, the comparison of differences between samples in different groups. These differences are compared based on the similarity of OTU sequences or the structure of the community. The results show that there are significant differences in the distribution and structure of intestinal flora between gallstone mice and normal mice. We used Bray-Curtis distance analysis (Supplementary Figure 2A), NMDS analysis (Supplementary Figure 2B), UPGMA repeated sampling reliability analysis (Supplementary Figure 2C), PCA (Supplementary Figure 2D), PCoA analysis (Supplementary Figure 2E) and sample hierarchical cluster analysis (Supplementary Figure 2F) to compare the flora of the C group and T group. The results show that there are significant differences in the distribution and structure of intestinal flora between gallstone mice and normal mice. The above data fully confirm that there is a serious imbalance in the gut microecology of gallstone mice.

**FIGURE 2:**
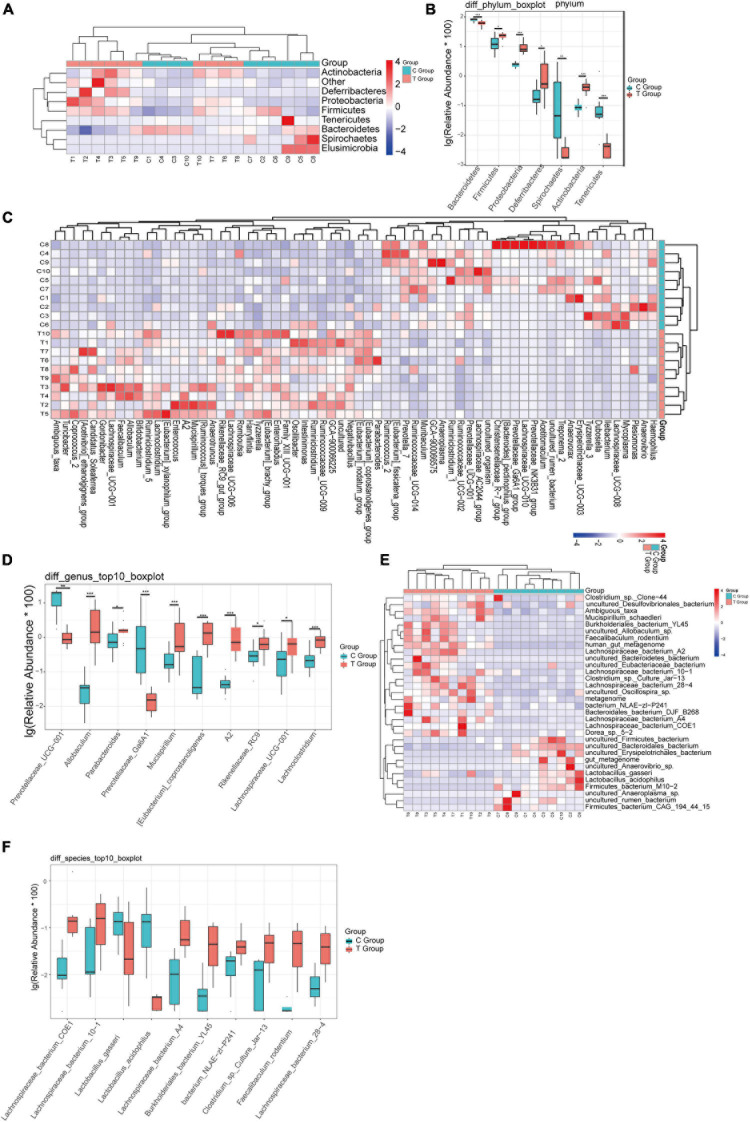
The difference in bacterial flora between the gallstone group and the control group was analyzed at different levels. **(A,B)** Analysis of changes in flora at the phylum level. **(C,D)** Analysis of changes in flora at the genus level. **(E,F)** Analysis of changes in flora at the species level. (Wilcoxon rank-sum test, ***p < 0.001, **p < 0.01, *p < 0.05).

To more clearly describe the changes in the intestinal flora of mice with gallstones, we made a comparison at the phylum, genus, and species levels. At the phylum level, we found that Proteobacteria, Actinobacteria, Deferribacteres, and Firmicutes were significantly increased in gallstone mice, while Bacteroidetes, Tenericutes, and Elusimicrobia were significantly reduced in gallstone mice (Figures 2A,B). At the genus level, 36 genera were significantly increased in gallstone mice, and 30 genera decreased significantly (Figure 2C). Eight of the top ten bacterial genera with the highest abundance of bacteria increased significantly in mice with gallstones (Figure 2D). At the species level, 21 species increased significantly in gallstone mice, and 11 species decreased significantly (Figure 2E). Figure 2F shows the top ten species, 8 of which were significantly increased in gallstone mice.

**FIGURE 3:**
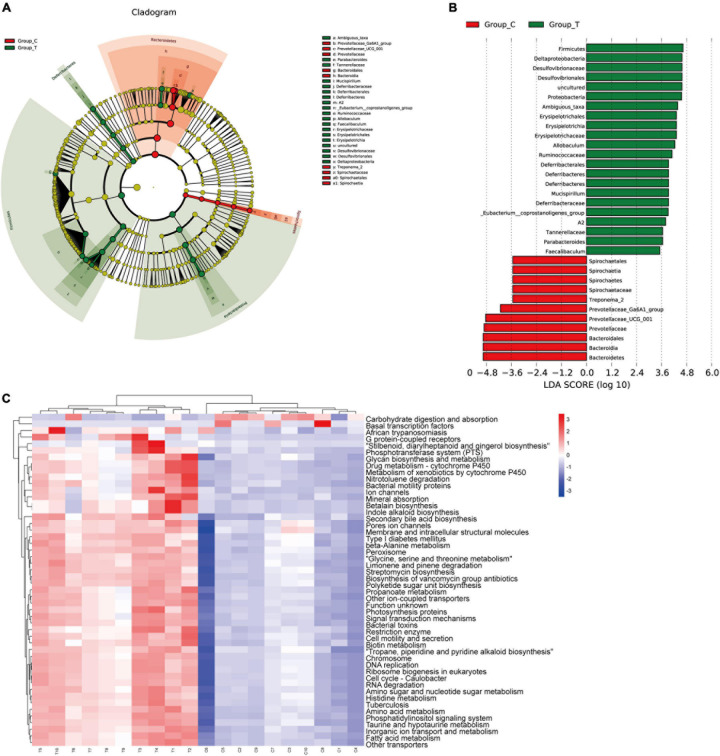
LEfSE analysis and PICRUSt analysis results. **(A)** The LEfSe cladogram in green shows the taxa enriched in the T group, and that in red shows the taxa enriched in the C group. The diameter of nodes is directly proportional to the relative abundance. **(B)** Taxonomic cladogram obtained using linear discriminant analysis (LDA) effect size (LEfSe) analysis and Mann–Whitney *U*-tests of the 16S sequences. LEfSe identified the taxa with the greatest differences in abundance between the T group and C group. At the phylum, class, order, family and genus levels, the control-enriched taxa are indicated by a positive LDA score (red), and T group-enriched taxa are indicated by a negative score (green). Only taxa meeting a significant LDA threshold value of > 2 are shown. **(C)** PICRUSt analysis results of predicted functional pathways in the gut microbiota.

To identify the specific communities and specific biomarkers in gallstone mice, we compared the compositions of the intestinal flora between the two groups using LEfSe analysis (Figures 3A,B). In total, LEfSe analysis revealed 33 discriminative features [phylum (5), class (5), order (5), family (7), and genus (11)]. At the phylum level, the abundance of Firmicutes was significantly enriched in the T group (LEfSe: *p* < 0.05, LDA>2), followed by Deferribacteres and Proteobacteria, whereas the abundance of Bacteroidia and Spirochaetia was enriched in the C group. At the family level, T group-enriched species included Tannerellaceae, Deferribacteraceae, Ruminococcaceae, Erysipelotrichaceae, and Desulfovibrionaceae, while Prevotellaceae and Spirochaetaceae were prevalent in the C group. At the genus level, 8 genera with significantly greater species abundances, including Ambiguous_taxa, Parabacteroides, Mucispirillum, A2, Eubacterium__coprostanoligenes_group, Allobaculum, and Faecalibaculum, were observed in the T group rather than in the C group, while Treponema_2, Prevotellaceae_UCG_001, and Prevotellaceae_Ga6A1_group were enriched in the C group.

To characterize the intestinal flora functional alterations after LD dietary intervention, we predicted functional composition profiles based on 16S rRNA sequencing data by performing phylogenetic reconstruction of unobserved states (PICRUSt) analysis. There were 215 different KEGG terms between the two groups. Figure 3C shows the top 50 with the most significant differences. The results suggested that many KEGG pathways, including ABC transporters, secondary bile acid biosynthesis, primary bile acid biosynthesis, lipid biosynthesis proteins, galactose metabolism, and glycerophospholipid metabolism, were significantly modulated after LD dietary intervention and were related to the formation mechanism of gallbladder stones. Some of the pathways were supported by subsequent LC/MS-based metabolomics analyses.

### Effects of LD Interventions on Liver Metabolomic Profiles

**FIGURE 4:**
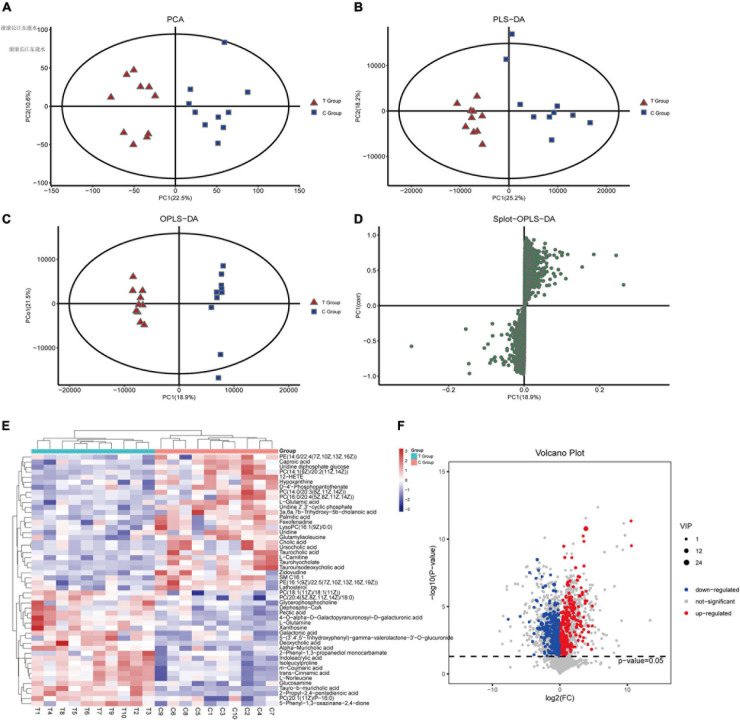
Effects of dietary interventions on liver metabolomic profiles. **(A)** PCA score plot of T and C groups. **(B)** PLS-DA score plot of T and C groups. **(C)** OPLS-DA score plot of T and C groups. **(D)** Plot based on PLS-DA score. **(E,F)** Significantly different metabolites and the expression levels of the top 50 differentially expressed metabolites with VIP values were analyzed by hierarchical clustering (VIP > 1, *p* < 0.05).

To evaluate changes in liver metabolic profiles caused by different dietary interventions, we performed non-targeted metabolomics profiling of paired liver samples, and 1271 compounds were identified. PCA (R2X = 0.546) and PLS-DA (R2X = 0.57, R2Y = 0.965, Q2 = 0.679) were performed to characterize the global metabolomics differences between the two groups. The results showed that the liver metabolism of gallstone mice was significantly different compared to that of the control group (Figures 4A,B). Next, supervised orthogonal partial least squares analysis (OPLS-DA) was used to distinguish the overall differences in metabolic profiles among groups and find the metabolites that were different between groups. As shown, the T group and C group could be separated into distinct regions according to their metabolic differences [OPLS-DA models: R2Y = 0.992 and Q2(cum) = 0.799 (Figure 4C)]. To prevent overfitting of the model, sevenfold cross validation and 200 response permutation tests (RPTs) were used to evaluate the quality of the model (OPLS-DA validation models: R2Y = 0.908 and Q2 = −0.461), showing that the OPLS-DA model possessed a satisfactory fit with good predictive power. The Splot-OPLS-DA chart shows the most significant metabolites, including taurohyocholate, PE and PC (Figure 4D).

To discover the different metabolites of biological significance, a combination of multidimensional analysis and single-dimensional analysis was applied. Finally, a total of 138 significantly changed metabolites (VIP > 1.0 and *p* < 0.05, Supplementary Material 3) were successfully identified between the two groups, as shown in the heatmap (Figures 4E,F). Further stratified analysis by metabolite categories showed that the 138 metabolites could be classified mainly into carboxylic acids and derivatives (31), steroids and steroid derivatives (24), organonitrogen compounds (17), glycerophospholipids (12), fatty acyls (12), pyrimidine nucleosides (9), purine nucleotides (7), benzene and substituted derivatives (6), organic sulfuric acids and derivatives (3), tetrapyrroles and derivatives (2), sphingolipids (2), other small molecules (11), and unclassified compounds (2). Compared with the C group, the levels of 57 metabolites increased in the T group. Among these metabolites, amino acids, peptides, and analog compounds increased in the T group, whereas bile acids, alcohols and derivative compounds decreased in the T group. We noticed that these metabolites included 3a,6b,7b-trihydroxy-5b-cholanoic acid, deoxycholic acid 3-glucuronide, chenodeoxycholic acid, nutritionolic acid, deoxycholic acid, tauro-b-muricholic acid, 2-propyl-2,4- pentadienoic acid, alpha-muricholic acid, glycerophosphocholine, galactonic acid, mesobilirubinogen, and N-alpha-acetyllysine. However, the concentration of primary bile acids (cholic acid, glycocholic acid, taurocholic acid) decreased in the T group. Supplementary Material 3 shows all of the different metabolites.

**FIGURE 5:**
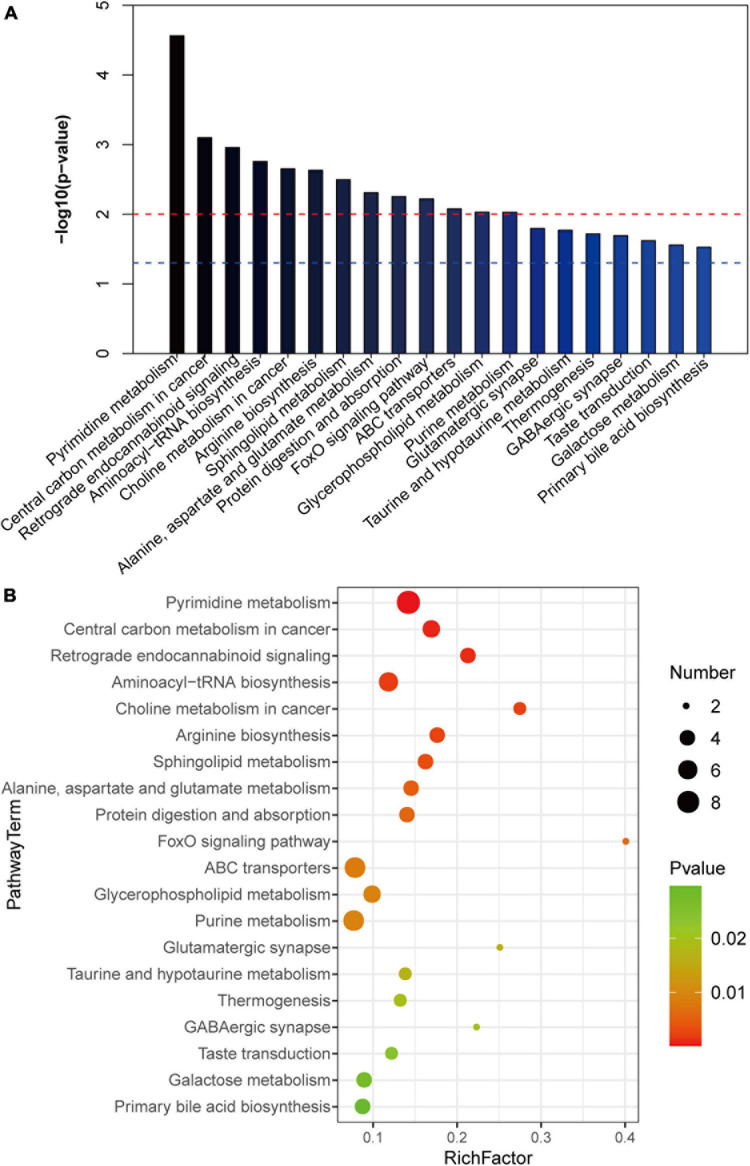
KEGG analysis of differential metabolites. **(A,B)** The top 20 metabolic pathways with the highest enrichment of differential metabolites (*p* < 0.05).

To further clarify the changes in liver metabolic pathways in mice with gallstones, we performed KEGG functional enrichment analysis on the differential metabolites. We show the top 20 metabolic pathways that are most meaningful (Figures 5A,B). We noticed that ABC transporters, glycerophospholipid metabolism, primary bile acid biosynthesis, galactose metabolism and aminoacyl-tRNA biosynthesis and the FoxO signaling pathway are related to the formation of gallstones.

### The Potential Link Between Intestinal Flora Imbalance and Liver Metabolism Changes Is Related to the Formation of Gallstones

**FIGURE 6:**
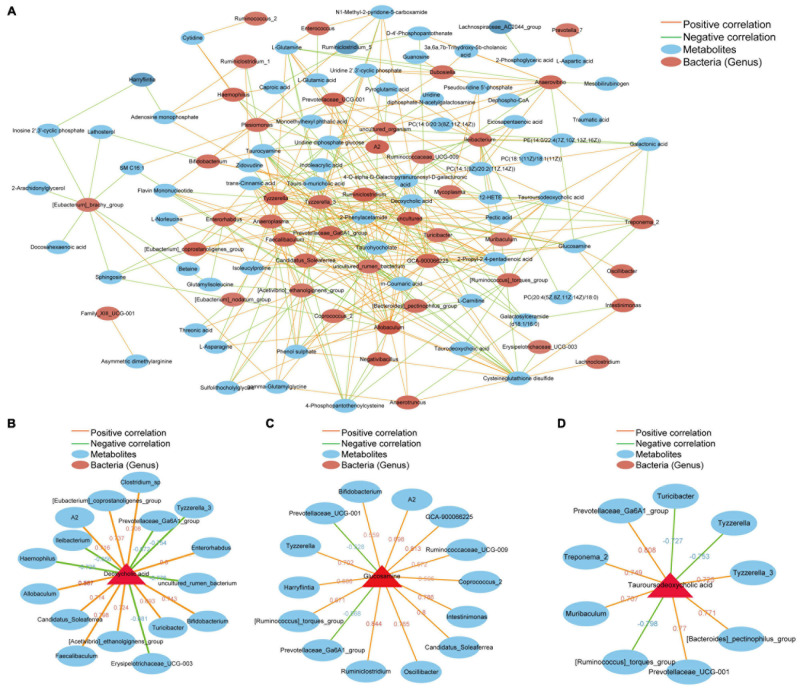
Potential correlations between the gut microbiota and liver metabolites. The red line represents a positive correlation, and the green line represents a negative correlation. The thickness of the line represents the correlation coefficient. **(A)** Correlation between 52 different intestinal bacterial genera and the top 100 metabolites. **(B)** Bacteria that are positively or negatively related to deoxycholic acid. **(C)** Bacteria that are positively or negatively related to glucosamine. **(D)** Bacteria that are positively or negatively related to tauroursodeoxycholic acid. (Student’s *t*-test, *p* < 0.05, Spearman correlation analysis, | correlation coefficient| ≥ 0.6).

To investigate the extent to which the altered gut microbiome was associated with liver metabolites in the host, we used Spearman’s correlation analysis to determine the covariation between the differential intestinal flora (52) and top 100 metabolites (Supplementary Material 4), which is presented in a heatmap (Figure 6A). A significant correlation (Student’s *t*-test, *p* < 0.05, | correlation coefficient| > 0.3) was identified between the changes in the gut microbiome and liver metabolite profiles. We focused on metabolites that are closely related to the formation of gallstones, including deoxycholic acid, asymmetric dimethylarginine, glucosamine and tauroursodeoxycholic acid. Deoxycholic acid (DCA) is elevated in the livers of mice in group T and is positively correlated with certain bacterial genera (Clostridium_sp, Allobaculum, Enterorhabdus, Faecalibaculum, Bifidobacterium, [Eubacterium]_coprostanoligenes_group, [Acetivibrio]_ethanolgignens_group, A2, Candreatus_Sobaclea). However, there was a negative correlation with other bacterial genera (Ileibacterium, Prevotellaceae_Ga6A1_group, Erysipelotrichaceae_UCG-003, Hemophilus, Tyzzerella_3, uncultured_rumen_bacterium) (Figure 6B). Asymmetric dimethylarginine increased in the T group and had a positive correlation with Family_XIII_UCG-001. Family_XIII_UCG-001 also increased significantly in the gallstone group (Figure 6A). Glucosamine has a positive correlation with A2 Intestinimonas, Ruminiclostridium, Oscillibacter, Bifidobacterium, Tyzzerella, GCA-900066225, Ruminococcaceae_UCG-009, [Ruminococcus]_torques_group, Coprococcus_2, Harryflintia, and Candidatus_Soleaferrea. However, it had a negative correlation with Prevotellaceae_UCG-001 and Prevotellaceae_Ga6A1_group (Figure 6C). Tauroursodeoxycholic acid was significantly reduced in mice with gallstones. TUDCA has a negative correlation with Muribaculum, Treponema_2, [Bacteroides]_pectinophilus, and Tyzzerella_3. Turicibacter, Tyzzerella, [Ruminococcus]_torques_group and TUDCA were negatively correlated and were significantly increased in the gallstone group (Figure 6D). Supplementary Material 5 shows the correlation analysis between different species and different metabolites at the species level. In general, these results indicate that changes in intestinal flora are related to changes in liver metabolites. We speculate that the interaction between these two factors may be an important target for driving or preventing gallbladder stones.

## Discussion

Numerous studies have demonstrated that the gut microbiome and its metabolites are widely involved in communication between the gut and the liver. Due to liver and intestinal flora coparticipation in the process by which bile acid and lipid metabolism regulate host immunity, the gut-liver axis has been found to be involved in the pathogenesis of liver and gall diseases such as non-alcoholic fatty liver disease (Boursier et al., 2016), primary biliary cholangitis (Tang et al., 2018), and liver cancer (Ma et al., 2018). The intestinal flora are associated with susceptibility to gallstone diseases (Wang et al., 2017, 2020). Our research results show that the intestinal flora of gallstone mice is seriously dysregulated. This finding is similar to previous studies investigating the gut microbiota of both gallstone patients (Wu et al., 2013; Wang et al., 2020) and mice (Fremont-Rahl et al., 2013). The results of the study showed that liver metabolism in mice with gallstones was also severely disturbed. We conducted a detailed analysis of these two factors.

In our study, deoxycholic acid (DCA) was significantly higher in the T group mice than in the C group mice. Studies have shown that DCA has a positive correlation with an increased prevalence of cholesterol gallstones (Thomas et al., 2005). DCA, as an FXR agonist (Wahlstrom et al., 2016), increases human gallbladder cholesterol saturation and bile acid hydrophobicity, and both decrease cholesterol solubility in bile and increase the risk of gallstone formation (Heaton, 2000). Members of the genus Clostridium show 7α-dehydroxylation enzymatic activity, which can convert host primary bile acids into secondary bile acids such as DCA (Berr et al., 1996). In our study, the abundance of Clostridium sp. in the T group was higher than that in the C group, and the abundance was positively correlated with DCA. Several studies have reported that patients or mice with gallstones have an increased relative abundance of the genus Clostridium (Wells et al., 2000; Wang et al., 2017). In addition, we found that DCA interacts and has a positive correlation with Allobaculum, Enterorhabdus, Faecalibaculum, Bifidobacterium, [Eubacterium]_coprostanoligenes_group, [Acetivibrio]_ethanolgignens_group, A2, Candidatus_Soleafer rea and Turicibacter but a negative correlation with Ileibacterium, Prevotellaceae_Ga6A1_group, Erysipelotricha ceae_UCG-003, Hemophilus, Tyzzerella_3, and uncultured_rumen_bacterium. Interestingly, as a probiotic, Bifidobacterium plays a therapeutic role in many diseases. Studies have shown that Bifidobacterium exerts a serum cholesterol-lowering effect and prevents hypercholesterolemia (Jones et al., 2013; Urdaneta and Casadesus, 2017). However, our study found that there was a significant increase in bifidobacteria in the gut of mice with gallstones. It is suggested that excessive probiotics (Bifidobacterium) may be related to the formation of gallstones. In fact, Clostridium, Bifidobacterium, and Enterorhabdus are involved in the metabolism of bile acids, and excess bile acids in the intestine disrupt enterohepatic circulation and may be involved in the formation of gallstones (Grigor’eva and Romanova, 2020).

Asymmetric dimethylarginine (ADMA), the methylated derivative of L-arginine, is a differential metabolite highly expressed in the T group. Increasing evidence has shown that the serum ADMA level has a positive correlation with insulin resistance (IR). Hepatic IR was found to be an independent risk factor for GSD (Biddinger et al., 2008; Chang et al., 2008); therefore, we infer that ADMA plays a promoting role in the process of gallstone development. ADMA is an endogenous inhibitor of nitric oxide (NO) synthetase, and endothelial dysfunction due to reduced bioavailability of nitric oxide (NO) is a risk factor for atherogenesis (Qin et al., 2021), myocardial infarction (Mannino et al., 2019), and chronic kidney disease (Shah et al., 2021). At the same time, studies have shown that GB hypomotility plays a key role in gallbladder (GB) normal relaxation (Chen et al., 1997; Swartz-Basile et al., 2000), while GB hypomotility is also a required factor for gallstone pathogenesis. Therefore, whether ADMA is friend or foe of gallstone formation requires further testing. In this study, ADMA was positively correlated with Family_XIII_UCG-001. Little information in the literature on Family XIII UCG-001 has shown that it is correlated with depression-like behavior in mice (Tian et al., 2019). The relationship between Family XIII UCG-001 and hepatic IR needs to be further tested.

Our study showed that glucosamine was a significantly elevated differential metabolite in the T group. Glucosamine is a precursor in the synthesis of mucin (Feller et al., 1990; Tailford et al., 2015). Mucin is well known to protect intestinal mucosal barrier function in the intestine, and abnormal mucin secretion appears in many tumor diseases and serves as a marker (Grondin et al., 2020; Paone and Cani, 2020). Previous studies have shown that mucin-4 is involved in gallstone formation (Di Ciaula et al., 2018; Hu et al., 2021). Specific bacteria and microbial products such as LPS can stimulate mucin secretion (Paone and Cani, 2020). Our research found that A2, Intestinimonas, Ruminiclostridium Oscillibacter, Bifidobacterium, Tyzzerella, GCA-900066225, Ruminococcaceae_UCG-009, [Ruminococcus]_torques_group, Coprococcus_2, Harryflintia, and Soleaferrea_idatus had a positive correlation with glucosamine. These bacterial genera are significantly increased in the gut of mice with gallstones. We speculate that these bacterial disorders may increase the production of glucosamine in the liver, thus creating conditions for the synthesis of mucin. This will undoubtedly accelerate the formation of gallstones. Interestingly, we found that two genera (Prevotellaceae_UCG-001 and Prevotellaceae_Ga6A1_group) had a negative correlation with glucosamine. They are all from the family Prevotellaceae and are reduced in the gallstone group. This suggests that the decreases in Prevotellaceae_UCG-001 and Prevotellaceae_Ga6A1_group may be related to the increase in glucosamine. These genera may have the potential to act as probiotics to prevent gallstones. Studies suggest that Tyzzerella accumulates in the intestines of obese children (Jaimes et al., 2021) and hyperlipidemic rats (Zhang et al., 2020). We hypothesized that intestinal flora are involved in the synthesis of mucin precursors, thereby affecting gallstone formation.

Tauroursodeoxycholic acid (TUDCA), which is a hydrophilic bile acid that inhibits intestinal inflammation, improves intestinal barrier function, and reduces the inflammation of the liver caused by LPS in blood, was significantly elevated in the C group (Wang et al., 2018). TUDCA can reduce the formation of gallstones by improving intestinal flora disorder in HFD-fed mice and inhibiting lipid absorption, intestinal cholesterol absorption and synthesis in the small intestine (Lu et al., 2020). Our results show a positive association between TUDCA and Prevotellaceae_UCG-001 (Figure 6D). A study showed that Prevotellaceae_UCG-001 was significantly enriched after inulin treatment, which helped to improve glucose and lipid metabolism (Song et al., 2019). Prevotellaceae, as a potential probiotic genera, is believed to be associated with the synthesis of short-chain fatty acids (SCFAs) (Xie et al., 2020; Zhu et al., 2020), which are important fuel for intestinal epithelial cells and are known to strengthen gut barrier function (Parada Venegas et al., 2019). The lack of SCFAs weakens their protective effect on the intestinal mucosal barrier, which may lead to increased levels of enterogenous endotoxin, further proving that the reduction in SCFA-producing bacteria may play an important role in the pathogenesis of NAFLD (Shen et al., 2017). Previous studies have shown that abnormal intestinal mucosal barrier function probably induces the formation of gallstones through a bacterial translocation mechanism (Su et al., 2009). We hypothesized that Prevotellaceae may play a protective role in inhibiting the formation of gallstones by regulating bile acid composition and protecting the intestinal barrier. Whether Prevotellaceae can be used as a non-invasive observation index for the detection of litholytic drugs remains to be further verified. In addition, we found that TUDCA had a negative correlation with Muribaculum, Treponema_2, [Bacteroides]_pectinophilus, and Tyzzerella_3. These bacteria were reduced in the gallstone group, and whether they can affect TUDCA as a target for preventing gallstones needs further exploration. Turicibacter, Tyzzerella, [Ruminococcus]_torques_group and TUDCA were negatively correlated, and they were significantly increased in the gallstone group. These bacteria may cause a decrease in TUDCA.

We noticed that the function of the differential flora was consistent with the metabolic pathway enriched by the liver’s differential metabolites, for example, taurine and hypotaurine metabolism, beta-alanine metabolism, pantothenate and CoA biosynthesis, D-glutamine and D-glutamate metabolism, purine metabolism, pyrimidine metabolism, glycerophospholipid metabolism, primary bile acid biosynthesis, alanine, aspartate and glutamate metabolism, glutamatergic synapse, aminoacyl metabolism, tRNA biosynthesis, ABC transporters, galactose metabolism, pentose and glucuronate interconversions. All of the pathways were supported by subsequent LC/MS-based metabolomics analyses. This suggests that the disordered flora caused liver metabolic disorders through these metabolic pathways, leading to gallstone formation.

## Conclusion

In summary, this study confirmed that mice with gallstones have severe intestinal flora imbalance, and these disordered flora will not recover without intervention. This becomes an important driving factor for the formation of gallstones. Through our research, biomarkers of gallstones were identified at the phylum (5), class (5), order (5), family (7), and genus levels. We predicted the functions of the differential flora, which provides an important reference for further intervention in the disease from the perspective of the flora. At the same time, we analyzed the liver metabolism of gallstone mice paired with their flora, and the results showed that there were 138 different metabolites between the two groups. We performed KEGG enrichment analysis on differential metabolites, and we were surprised to find that the metabolic pathways enriched by these differential metabolites were highly consistent with the function of the disordered flora. Therefore, we conducted an association analysis of the two factors, and we constructed a network of relationships between disordered bacteria and disordered liver metabolites. This provides a basis for the establishment of the intestine-liver axis in gallstone disease. Furthermore, this research provides a theoretical basis for the research and development of probiotics and prebiotics. We focused on the analysis of the relationship between deoxycholic acid, asymmetric dimethylarginine, glucosamine, tauroursodeoxycholic acid, and the disordered flora. It is important that DCA is a contributing factor to the formation of gallstones, but Bifidobacterium, a well-known probiotic, also has a positive correlation and increases significantly in the gallstone group. The Family XIII UCG-001-ADMA axis may promote the occurrence and progression of gallstones. In the pathogenesis of gallstones, glucosamine is a risk factor that increases in the gallstone group, while TUDCA is a protective factor that decreases in the gallstone group. Interestingly, we found that Prevotellaceae decreased in the gallstone group and had a positive correlation with glucosamine but a negative correlation with TUDCA. Therefore, we speculate that Prevotellaceae can be used as a probiotic to treat gallstones.

## Data Availability Statement

The data presented in the study are deposited in the NCBI BioProject accession number is: PRJNA736820 and in metabolights accession number is: MTBLS2945.

## Ethics Statement

The animal study was reviewed and approved by the Institutional Animal Care and Use Committee of the First Affiliated Hospital of Harbin Medical University.

## Author Contributions

YC and QW participated in the analysis of data, writing of the manuscript, and specimen collection. WG and BM participated in data collection and analysis. DX and CH participated in the study conception and supervision and data analysis and manuscript editing. All authors read and approved the final version of the manuscript.

## Conflict of Interest

The authors declare that the research was conducted in the absence of any commercial or financial relationships that could be construed as a potential conflict of interest.

## Publisher’s Note

All claims expressed in this article are solely those of the authors and do not necessarily represent those of their affiliated organizations, or those of the publisher, the editors and the reviewers. Any product that may be evaluated in this article, or claim that may be made by its manufacturer, is not guaranteed or endorsed by the publisher.

## Footnotes

1
http://www.genome.jp/kegg

## Supplementary Material

Supplementary Figure 1 Changes in gut microbiota diversity and composition after dietary intervention. **(A)** The CHAO1 index of gallstone model mice was significantly reduced. **(B)** The Good’s coverage index of gallstone model mice increased significantly. **(C)** The Specaccum species accumulation curve tends to gradually flatten, indicating that samples are sufficient, and its samples can reflect the richness of species. **(D)** Rank abundance curve reflects the abundance and uniformity of species in samples.

Supplementary Figure 2 Beta diversity is the degree of diversity between biological environments. **(A)** According to the distance matrix obtained by the Bray-Curtis distance algorithm, the matrix is clustered hierarchically, and the distance of the sample branches can be clearly seen. The bluer the color, the closer the distance between the samples, and the higher the similarity; the redder the color, the further the distance. **(B)** NMDS analysis. The abscissa (NMDS1) and the ordinate (NMDS2) are two sorting axes. Each point in the figure represents a sample. The same color is the same group. Similar samples will gather together. If the difference between the samples is large, the distance is far away. **(C)** UPGMA repeated sampling reliability analysis. The red part represents a reliability of 75–100%; the yellow part is 50–75%; the green part is 25–50%; the blue part is less than 25%. **(D)** PCA: The more similar the sample composition, the closer the distance reflected in the PCA chart; the more different the sample composition, the farther the distance reflected in the PCA chart. Based on the analysis results of the Bray-Curtis distance matrix, PCoA can be used to observe the differences between individuals or groups. Each point in the figure represents a sample. The same color is the same group. If the samples of the same group are closer together and further away from other groups, this shows that the grouping effect is good. **(F)** Sample hierarchical cluster analysis: The closer the branch distance, the more similar the samples.

Supplementary Table 1 An excel sheet of all differential metabolites detected in liver data (Wilcoxon rank-sum test, ^∗∗∗^*p* < 0.001, ^∗∗^*p* < 0.01, ^∗^*p* < 0.05).

Supplementary Table 2 Data table of the relationship between sequencing and differential metabolites (Wilcoxon rank-sum test, ^∗∗∗^*p* < 0.001, ^∗∗^*p* < 0.01, ^∗^*p* < 0.05; Spearman correlation analysis, | correlation coefficient| ≥ 0.6).

Supplementary Table 3 Correlation analysis between different species and different metabolites at the species level (Wilcoxon rank-sum test, ^∗∗∗^*p* < 0.001, ^∗∗^*p* < 0.01, ^∗^*p* < 0.05; Spearman correlation analysis, | correlation coefficient| ≥ 0.6).
