# Supplementary figures and images for: Changes and Correlations of the Intestinal Flora and Liver Metabolite Profiles in Mice With Gallstones
Source: Front Physiol. 2021 Aug 19;12:716654. doi: 10.3389/fphys.2021.716654 (PMC8416897; doi:10.3389/fphys.2021.716654)

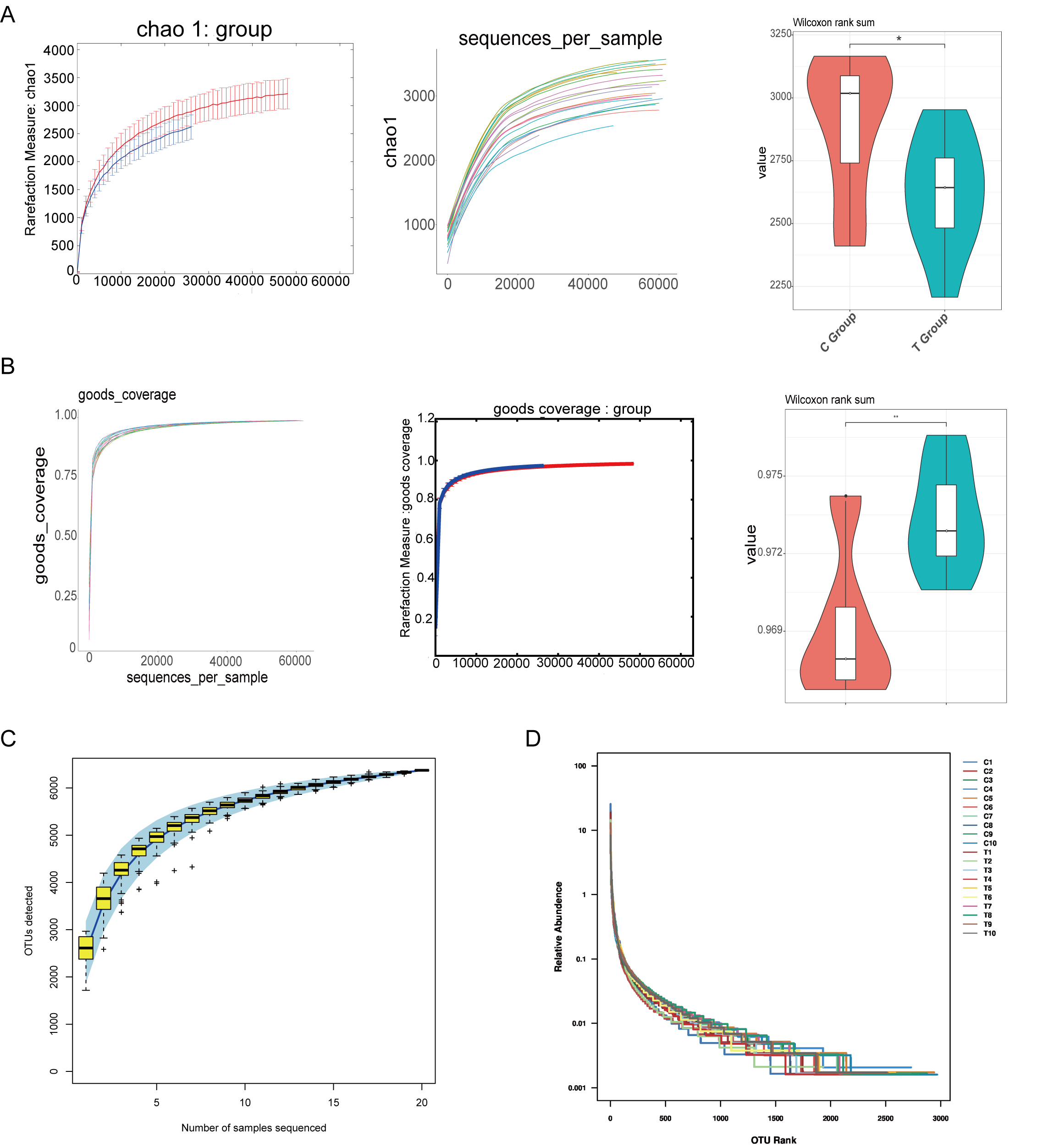

Supplement: Supplementary Figure 1 — Changes in gut microbiota diversity and composition after dietary intervention. (A) The CHAO1 index of gallstone model mice was significantly reduced. (B) The Good’s coverage index of gallstone model mice increased significantly. (C) The Specaccum species accumulation curve tends to gradually flatten, indicating that samples are sufficient, and its samples can reflect the richness of species. (D) Rank abundance curve reflects the abundance and uniformity of species in samples. [file Image_1.TIF]

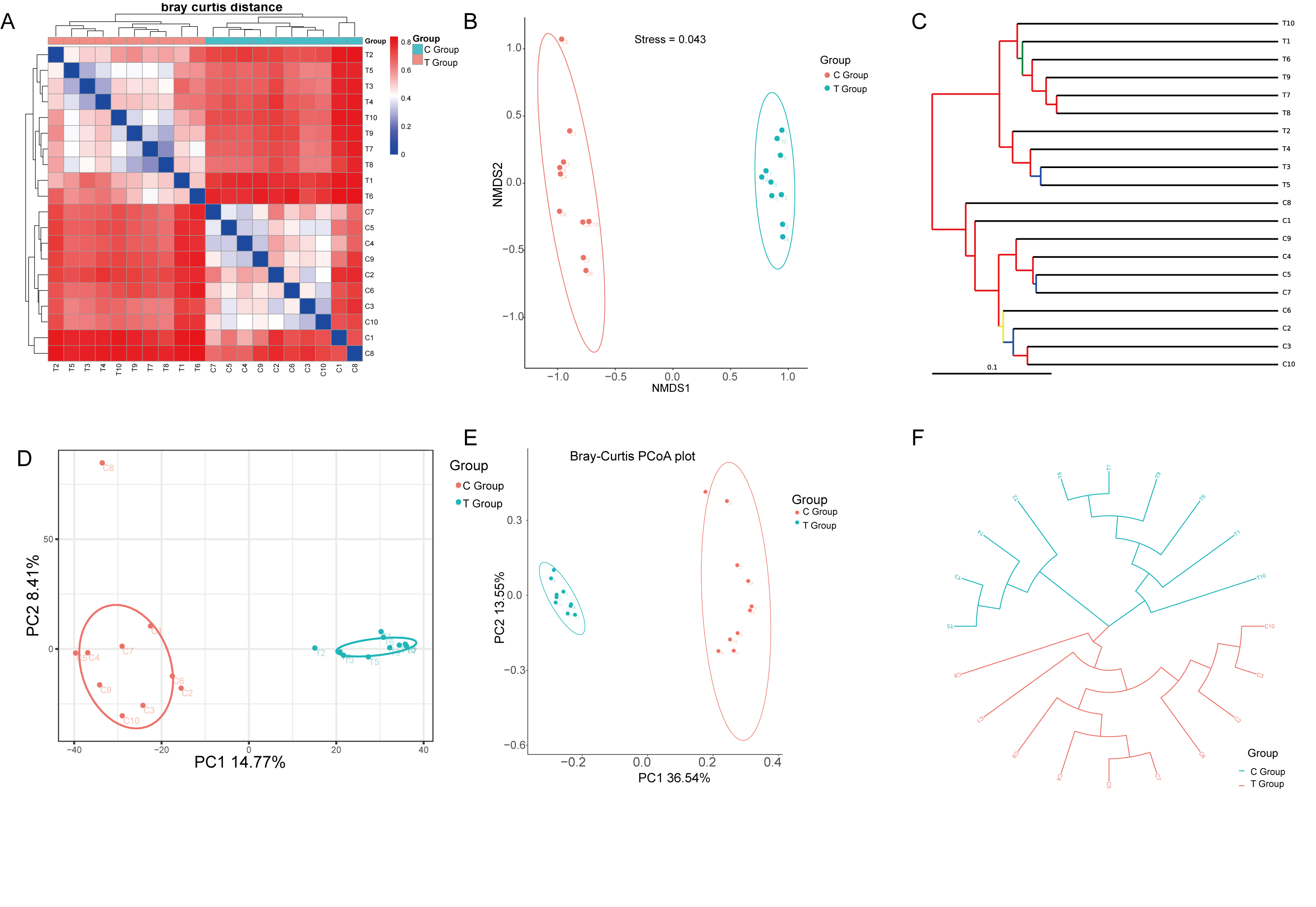

Supplement: Supplementary Figure 2 — Beta diversity is the degree of diversity between biological environments. (A) According to the distance matrix obtained by the Bray-Curtis distance algorithm, the matrix is clustered hierarchically, and the distance of the sample branches can be clearly seen. The bluer the color, the closer the distance between the samples, and the higher the similarity; the redder the color, the further the distance. (B) NMDS analysis. The abscissa (NMDS1) and the ordinate (NMDS2) are two sorting axes. Each point in the figure represents a sample. The same color is the same group. Similar samples will gather together. If the difference between the samples is large, the distance is far away. (C) UPGMA repeated sampling reliability analysis. The red part represents a reliability of 75–100%; the yellow part is 50–75%; the green part is 25–50%; the blue part is less than 25%. (D) PCA: The more similar the sample composition, the closer the distance reflected in the PCA chart; the more different the sample composition, the farther the distance reflected in the PCA chart. Based on the analysis results of the Bray-Curtis distance matrix, PCoA can be used to observe the differences between individuals or groups. Each point in the figure represents a sample. The same color is the same group. If the samples of the same group are closer together and further away from other groups, this shows that the grouping effect is good. (F) Sample hierarchical cluster analysis: The closer the branch distance, the more similar the samples. [file Image_2.TIF]
